# Acute objective and subjective intoxication effects of legal-market high potency THC-dominant versus CBD-dominant cannabis concentrates

**DOI:** 10.1038/s41598-021-01128-2

**Published:** 2021-11-05

**Authors:** M. L. Drennan, H. C. Karoly, A. D. Bryan, K. E. Hutchison, L. C. Bidwell

**Affiliations:** 1 Department of Psychology, Colorado State University, Fort Collins, CO 80525 USA; 2 Institute of Cognitive Science, University of Colorado Boulder, Boulder, CO 80309 USA; 3 Department of Psychology and Neuroscience, University of Colorado Boulder, Boulder, CO 80309 USA

**Keywords:** Neuroscience, Physiology, Psychology

## Abstract

As the market for cannabis concentrate products grows, the lack of research regarding the effects of concentrated THC and CBD becomes more glaring. The present study analyzes cannabinoid blood levels and subjective outcomes of physical sensation and affective state after ad libitum use of legal-market concentrate products. Recreational cannabis users were randomly assigned to THC- or CBD-dominant concentrate products, completing a baseline session, and an experimental mobile laboratory session consisting of timepoints before, immediately after, and one-hour after concentrate use. THC-dominant concentrates induced higher intoxication, and higher ratings of drug effect and drug liking than the CBD-dominant concentrate. Both products induced immediate feelings of elation, diminishing over the subsequent hour. Subjective outcomes in the CBD-dominant group revealed immediate decreases in tension and anxiety relative to pre-use, while the THC-dominant group only saw significant decreases in anxiety after one hour. Paranoia spiked immediately post-use in THC-dominant concentrate users, returning to baseline within an hour. Overall, the CBD-dominant concentrate invoked positive mood effects, lower intoxication and an absence of undesirable effects experienced with the THC-dominant concentrate, potentially mitigating negative effects when combined. Results support the need for further investigation into harm-reduction potential of concentrated CBD when used alone and with THC.

## Introduction

Of the hundreds of phytocannabinoids present in the *Cannabis sativa* plant, the most well-known is delta-9-tetrahydrocannabinol (THC). THC is the primary psychoactive component found in cannabis and is responsible for the euphoric high that many users seek. As accessibility to cannabis products increases in the United States due to legalization in many states, high potency THC products are growing in popularity^1^. These concentrated products contain 69% THC on average^2^, although potency can reach over 90% in some cases^1,3–6^. In contrast, cannabis flower typically contains between 10–25% THC (19% on average)^1^. Cannabis concentrates are made with cannabinoids that are extracted from the plant using various methods^7,8^. The final product comes in multiple forms including oil, wax, and hash, and is most commonly consumed via inhalation with a water pipe or vaporizer^9^.

Highly potent THC products may mean less product is required to achieve a quick and robust effect. Use of THC results in subjective reports of euphoric high, drug liking and increased positive mood^10,11^. THC also plays a role in the drug reward properties underlying the abuse liability of cannabis^12^. These rewarding effects can be accompanied by subjective reports of negative affect such as increased paranoia and anxiety^13^, which may worsen with high potency^14^. Cognitively, THC intoxication involves impairment to executive functioning, attention, impulse control and psychomotor function^8,15–17^. A recent publication by our group highlights some of the first data on the acute effects after use of high-potency THC concentrate products, showing increases in intoxication levels and cannabis effects from pre- to post-use, as well as higher plasma THC content when compared with cannabis flower^18^. However, data are limited on the impacts of these more potent forms and a growing concern is the potential risk of high THC exposure due to links with greater intoxication, affective problems, and long-term problem use^19,20^.

Second to THC, cannabidiol (CBD) is another abundant and commonly extracted cannabinoid found in legal-market cannabis. While THC comprises the majority of the concentrate market, high-concentrate CBD products represent a subset of products available. Unlike THC, CBD is thought to be non-intoxicating and does not impair cognition^21,22^. Despite limited research on its efficacy as a treatment, it is frequently marketed to recreational and medical users to treat an array of conditions such as pain^23,24^, with anxiety being a primary motivator for use of CBD products^25–28^. Finally, emerging but mixed evidence suggests that CBD may alter the effects of THC^3,29^, including potential mitigation of negative effects such as paranoia and anxiety^27^. Given that research has primarily highlighted differences in effects of THC and CBD alone, further studies are needed to explicitly test the interaction of these cannabinoids.

Due to federal restrictions preventing researchers from conducting highly controlled experimental studies using these legal-market products, empirical data regarding the acute effects of concentrate products and the effects of phytocannabinoids in their concentrated form are limited. Our group recently published a study on the acute effects of cannabis concentrates^16^, however no studies to our knowledge have compared the effects of THC-dominant and CBD-dominant concentrates. Currently, there is a significant gap in the literature regarding the effects of CBD-dominant concentrates, and the present study aimed to take a first step to fill this gap. Using an observational design involving self-administration of legal-market cannabis, the present study aimed to analyze cannabinoid blood levels, as well as explore subjective outcomes of physical sensation and affective state after self-administration of legal-market concentrated products with either high THC or high CBD content. It was expected that highly concentrated THC products would produce intoxication and enhance elation (i.e. positive mood), while products containing concentrated CBD with low THC levels would be anxiolytic and less intoxicating.

## Methods

### Participants

This study was approved by the University of Colorado Boulder Institutional Review Board (IRB Protocol Number 16–0768) and was carried out in accordance with the Declaration of Helsinki. Similar naturalistic administration methods have been reported in our previous work^17^. For this study protocol, participants were recruited using social media posts and mailed flyers. Eligibility screening was completed via telephone by trained research staff. Inclusion criteria were: (1) between 21 and 70 years of age; (2) cannabis use at least four times in the past month; (3) experience with the highest potency of cannabis that could be assigned in the study (i.e., 88% THC); (4) no other non-prescription drug use in the past 60 days; (5) no daily tobacco use; (6) two or fewer drinking occasions per week, with three or fewer drinks per occasion, at the time of screening; (7) not pregnant or trying to become pregnant; and (8) no current or history of any psychotic or bipolar disorders. Fifty-four participants (28 male, 26 female) provided informed consent and had complete data for the baseline and experimental appointments that were included in analyses.

### Appointments

#### Baseline appointment

The first appointment was conducted at the Center for Innovation and Creativity (CINC) where participants provided informed consent, were screened via urinalysis for pregnancy (if female) and the presence of alcohol, sedatives, cocaine, opiates, and amphetamines. Participants were asked not to use cannabis for 72 h prior to the baseline appointment. Participants also completed measures of demographics, lifestyle, substance use, medical history, subjective experience and several cognitive tests, and provided a baseline blood draw.

Participants were then randomly assigned to one of two potency conditions using a random assignment table generated by the study statistician, and given instructions to purchase 1 g of their assigned cannabis concentrate product (e.g., THC-dominant or CBD-dominant concentrate) at a local, study-partnered cannabis dispensary. The THC-dominant concentrate product contained 84.99% THC + THCa and < 1% CBD. The CBD-dominant concentrate product contained 74.7% CBD, 4.1% CBDa and 4.5% THC + THCa. In line with the naturalistic design of the study, the products were selected to reflect commonly used forms of concentrates on the legal-market and the average range of THC and CBD found in concentrate products on the Colorado market^3^. For example, it is common for high CBD concentrates on the legal market to contain some level of THC, due to extraction methods which result in low levels of other cannabinoids^8^. The potency of all study products was labeled according to testing in an International Organization of Standards (ISO) 17,025 accredited laboratory.

#### Experimental appointment

Participants were asked to use their cannabis concentrate as they normally would during the five-day period between the baseline and experimental appointment. On the fifth day, which was the day of the experimental appointment, our mobile laboratory was parked outside the participant’s residence for the duration of the session. Participants were instructed to abstain from using any form of cannabis that day prior to their appointment. At the start of the session, participants completed primary outcome measures (described below) prior to using their cannabis concentrate (pre-use). Next, they returned to their place of residence to self-administer their assigned cannabis product. They were asked to use as much of the product as they would typically use.

Participants were also instructed to weigh their cannabis concentrate product both before and after use with a study provided electronic scale to provide a measure of amount (mg) of cannabis concentrate used during ad libitum administration. Immediately after using their cannabis concentrate product, participants returned to the mobile lab (with the time spent away from the mobile lab being recorded), and completed the same outcome measures (immediate post-use). Participants remained in the mobile lab until one hour after using their product (1-h post-use) and completed the same outcome measures a final time. Thus, participants were assessed at three timepoints: pre-use, immediate post-use, and 1-h post-use. After this final data collection timepoint, participants returned to their place of residence and the experimental session was complete.

### Baseline demographic and substance use variables

#### Cannabis use disorder symptoms

Cannabis Use Disorder symptoms were assessed using the Marijuana Dependence Scale (MDS)^30^, an 11-item self-report measure developed based on dependence criteria found in the Diagnostic and Statistical Manual of Mental Disorders (DSM; α = 0.80).

#### Alcohol use disorders identification test (AUDIT)

As cannabis use often overlaps with other substance use^31^, participants completed the self-report AUDIT^32^. The range of possible scores is 0–40, with a score of 8 or more indicating harmful or hazardous drinking (α = 0.75).

#### Beck’s depression inventory II (BDI) and Beck’s anxiety inventory (BAI)

As a measure of describing trends in mental health within the sample, the BDI and BAI were administered. The BDI (α = 0.90) and BAI (α = 0.91) are both 21-item self-report scales that appraise depression and anxiety symptoms (respectively) over the two weeks prior to completing the assessment. Scores are summed (ranging from 0–63) and levels of depression or anxiety are determined to be minimal, mild, moderate, or severe^33,34^.

#### Timeline follow back (TLFB)

Participants completed a calendar-assisted, researcher administered TLFB that queried their drug use over a 30-day retrospective timeframe^35^. The current study includes TLFB measures of cannabis flower use, cannabis concentrate (dab) use, orally-ingested cannabis use and alcohol use. Participants also completed a TLFB for the 5 days prior to van session during which ad libitum concentrate use was monitored.

### Primary outcome measures

#### Blood cannabinoids

Thirty-two mL of venous blood were collected by a certified phlebotomist through venipuncture of a peripheral arm using standard, sterile phlebotomy techniques and plasma was separated from erythrocytes. Quantification of CBD, THC, and its primary metabolites THC-COOH and 11-OH-THC was conducted using validated high-performance liquid chromatography/mass-spectroscopy (HPLC–MS/MS) (API5500) in MRM mode.

#### Rewarding and subjective drug effects

Participants’ subjective cannabis intoxication was assessed using the 12-item Addiction Research Center Inventory-Marijuana (ARCI-M) effects scale^36^. Three additional items assessed cannabis intoxication: “mentally stoned” (5-point scale), “physically stoned” (5-point scale), and “feel high” (10-point scale). Scales were normalized and responses were averaged to create a composite intoxication score (α = 0.88)^3,18^. A modified version of the Profile of Mood States (POMS) questionnaire assessed state affect^18,37^. POMS items were rated on a 5-point Likert scale ranging from not at all (1) to extremely (5) and averaged to create subscales of Tension (“nervous”, “anxious”, “unable to relax”, “shaky/jittery”; α = 0.82) and Elation (“joyful”, “euphoric”, “elated”, “cheerful”; α = 0.79). As a measure of Drug reward, drug liking was assessed with a single item from the DEQ (“*do you like any of the effects you’re feeling*?”) ranging from not at all (1) to a lot (5)^38^.

### Analysis

**Figure 1:**
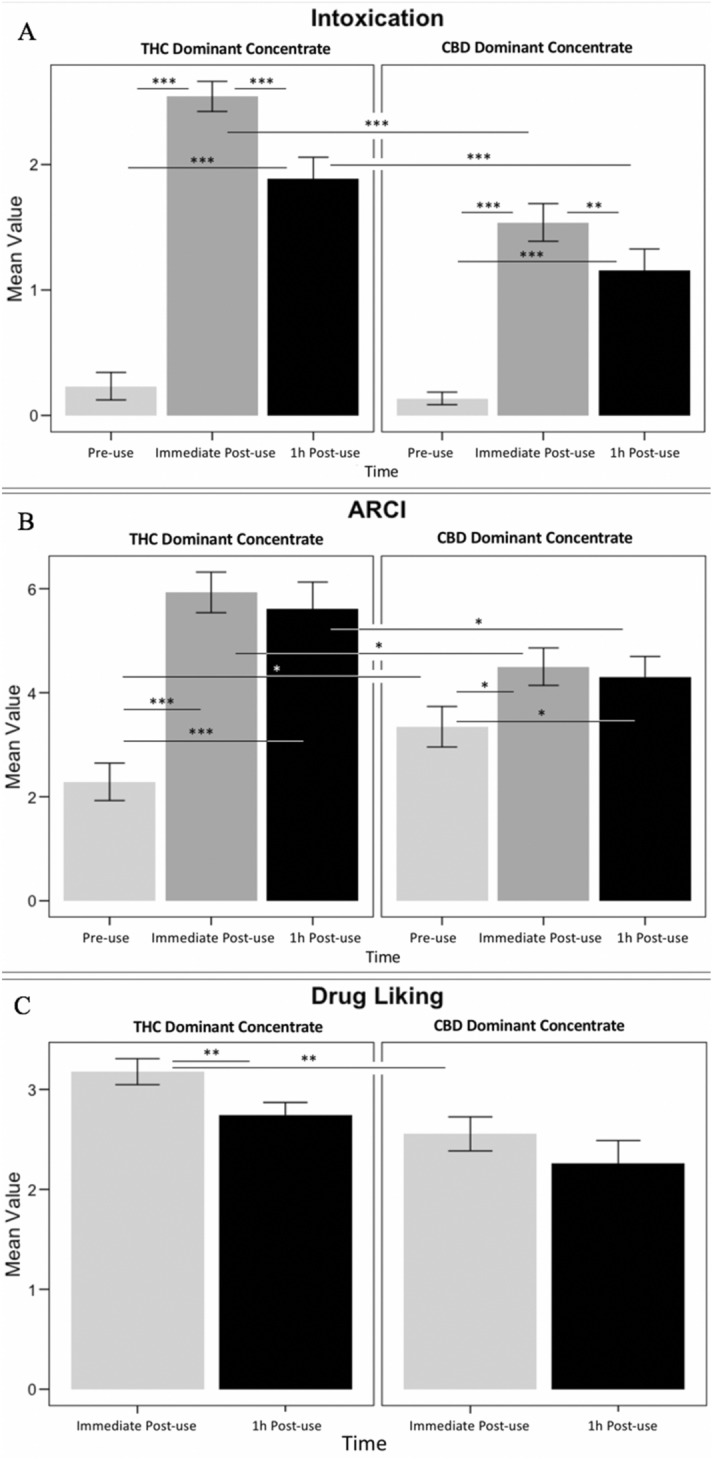
Subjective drug effects; within group significant differences were determined by paired t-tests and between group differences were determined by independent t-tests for the purpose of denoting differences in timepoints visually. (**A**) Composite self-report intoxication scale, (**B**) ARCI drug effect, (**C**) DEQ drug liking measure. (*) indicates significance at p < 0.05; (**) indicates significance at p < 0.01; (***) indicates significance at p < 0.001.

**Figure 2:**
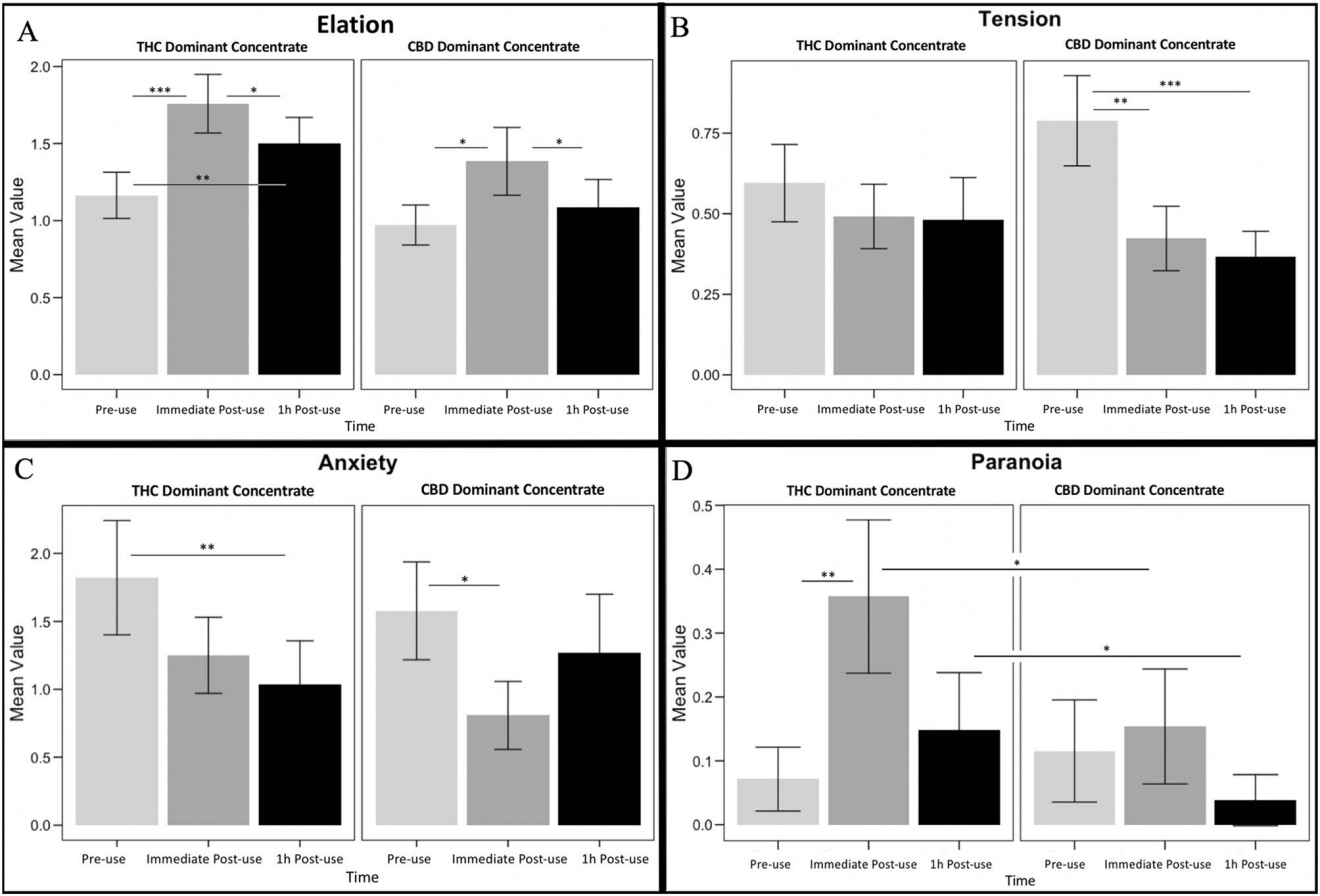
Subjective affect measures by timepoint and treatment group. Within group significant differences were determined by paired t-tests, and between group differences were determined by independent t-tests for the purpose of denoting differences in timepoints visually. (**A**) POMS elation (vigor) subscale; (**B**) POMS tension subscale; (**C**) anxiety measure from Marijuana Craving Questionnaire; D) POMS single item “paranoid”. (*) indicates significance at p < 0.05; (**) indicates significance at p < 0.01; (***) indicates significance at p < 0.001.

Between-group differences over time were analyzed using Repeated Measures Analyses of Covariance (RM ANCOVA) in R. Sex was included as a covariate in all models due to potential differences between males and females in cannabis concentrate use^39^ and functioning of the endocannabinoid system^40–42^. Within-group differences were assessed by paired t-tests and between group differences were determined by independent t-tests in order to visualize significant changes by time (Figs. 1, 2). Figures were created in R using the ggplot2 package.

## Results

### Baseline descriptive information

Eighty-one participants were initially enrolled in the study. However, due to COVID-19 and the associated laboratory shut-down in March, 2020, 17 individuals were not given the opportunity to complete the study. Thus, individuals who did not complete the van appointment and those with missing blood cannabinoid data at more than one timepoint (n = 6) were excluded. Additionally, 4 individuals with baseline blood levels of THC above 3 standard deviations from the mean were excluded, as this suggested they did not comply with the pre-baseline abstinence instructions. Fifty-four participants who took part in both baseline and experimental sessions were included in analyses after these exclusions: 28 participants (51.9%) were assigned to the THC-dominant concentrate condition (mean [SD] age 29.86 [8.7] years; 42.9%women) and 26 were assigned to the CBD-dominant concentrate condition (mean [SD] 29.88 [10.5] years; 53.8% women). Ages of participants ranged from 21 to 60 in both groups. No significant differences were noted in demographic variables between groups at baseline (Table 1), indicating that equivalent groups were created.

**Table 1:** Sample demographic, cannabis use, and other health characteristics by overall sample and concentrate assignment.

**Characteristic**	Overall (*n* = 54)	THC Concentrate (*n* = 28)	CBD Concentrate (*n* = 26)
**Demographics**			
Age, y, mean (SD)	29.87 (9.5)	29.86 (8.7)	29.88 (10.5)
Women, No. (%)	26 (48.1)	12 (42.9)	14 (53.8)
Marital status, married, No. (%)	11 (20.4)	5 (17.9)	6 (23.1)
Education, bachelor’s degree or higher, No. (%)	24 (44.4)	12 (42.9)	12 (46.2)
Employment, full-time No. (%)	30 (55.6)	18 (64.3)	12 (46.2)
Race, white, No. (%)	39 (72.2)	18 (64.3)	21 (80.8)
**Cannabis history and use measures, mean (SD)**			
Days of concentrate use in past 30 d	17 (11.9)	17.5 (11.9)	16.5 (12.2)
Days of flower use in past 30 d	20.5 (11.3)	20.3 (11.9)	20.7 (10.4)
Baseline plasma THC, μg/mL	5.35 (6.4)	6.91 (7.8)	3.73 (4.1)
Baseline plasma 11-OH-THC, μg/mL	1.75 (3.3)	2.51 (0.8)	0.95 (1.4)
Baseline plasma THC-COOH, μg/mL	57.75 (90.2)	68.19 (83.2)	46.88 (97.4)
Baseline plasma CBD, ng/mL	0.76 (2.6)	1.08 (3.6)	0.44 (0.7)
**Other substance use and psychological factors**			
Days of alcohol use in past 30 d, mean (SD)	9.09 (7.66)	8.43 (7.24)	9.84 (8.19)
Prescribed psychiatric medications^a^, No. (%)	2 (3.4)	2 (6.3)	0
Age of regular cannabis use onset, mean (SD)	19.3 (8.0)	19.6 (9.2)	19 (6.7)
Baseline Depression (BDI total), mean (SD)	9.33 (7.4)	8.75 (6.1)	9.96 (8.8)
Baseline Anxiety (BAI total), mean (SD)	9.04 (8.7)	8.36 (9.1)	9.77 (8.3)
AUDIT total, mean (SD)	5.48 (3.5)	5.36 (3.9)	5.62 (3.2)
MDS total, mean (SD)	3.30 (3.09)	3.21 (3.45)	3.38 (2.71)
**Cannabis use during ad libitum administration**			
Dabs/hits taken during experimental appointment, mean (SD)	3.16 (3.36)	2.88 (0.52)	3.46 (0.77)
Mode of ad libitum administration, No. (%)			
Glass rig (dab)	36 (66.7)	19 (67.9)	17 (65.4)
Glass tube (dab)	12 (22.2)	6 (21.4)	6 (23.1)
Hash pen (vape)	5 (9.3)	3 (10.7)	2 (7.7)

^a^The 2 participants (2.5%) endorsing psychiatric medications reported taking prescriptions for attention-deficit/ hyperactivity disorder (ADHD).

No significant differences were found to exist between conditions in any measure.

### Between baseline and experimental appointment: 5-day TLFB

Data on cannabis and alcohol use behavior before the experimental appointment is presented to show participants’ typical patterns of use and similarity across groups. As shown in Table 2, in the 5 days prior to the van session, participants used cannabis an average of 3.61 (SD = 1.12) days, and concentrates an average of 2.93 (SD = 1.5) days. Participants across conditions also used alcohol an average of 1.29 (SD = 1.23) days, and used alcohol and cannabis together an average of 1.05 (SD = 1.17) days. Pre-use plasma THC content did not significantly differ between groups given a 95% confidence interval (*p* = 0.06). Although the difference approached significance, indicating that the CBD-dominant group had used THC within the week prior to the van session, it was not clinically meaningful in the scope of our within-subjects analyses.

**Table 2:** Descriptive data on typical patterns of cannabis and alcohol use prior to the experimental session data.

5-day TLFB (Prior to van session)	Overall	THC Concentrate	CBD Concentrate	
Days of cannabis use, mean (SD)	3.61 (1.12)	3.79 (0.82)		3.41 (1.37)
Days of concentrate use, mean (SD)	2.93 (1.50)	3.28 (1.25)		2.56 (1.67)
Number of concentrate dabs/hits, mean (SD)	10.39 (9.53)	11.17 (7.57)		9.56 (11.36)
Number of concentrate grams used, mean (SD)	0.81 (1.04)	0.97 (0.96)		0.63 (1.11)
Days of alcohol use, mean (SD)	1.29 (1.23)	1.17 (1.07)		1.41 (1.39)
Drinks per day, mean (SD)	2.25 (2.91)	1.88 (1.88)		2.65 (3.71)
Days of alcohol & cannabis co-use, mean (SD)	1.05 (1.17)	1.07 (1.03)		1.04 (1.32)

### Mobile laboratory appointment: cannabinoid blood levels and validation of concentrate assignment

Participants in both groups used similar amounts of cannabis by weight during ad libitum administration (an average of 0.12 g concentrate per session across conditions: 0.13 g in the CBD-dominant condition, and 0.10 g in the THC-dominant condition; see Table 1). On average, participants spent 16.2 min (SD = 6 min, range = 6–36 min) away from the mobile laboratory during this time. THC and 11-OH-THC levels exhibited significant differences by timepoint, with levels peaking immediately post-use and dropping thereafter in both groups (Table 3). Further, the plasma levels in the respective groups indicate compliance with assigned treatment group (i.e., participants in the CBD group used a CBD product, and those in the THC group used a THC product). Participants in the THC-dominant concentrate group showed higher THC and 11-OH-THC levels, while the CBD-dominant concentrate group displayed a significant spike in CBD levels only immediately post-use.

**Table 3:** Means and standard errors of cannabinoid plasma level by timepoint and treatment group.

	Mean (SE)					
	THC Concentrate			CBD Concentrate		
	Pre-use	Immediate Post-use	1 h Post-use	Pre-use	Immediate Post-use	1 h Post-use
**Cannabinoids**						
THC, μg/mL	15.42 (6.68)	238.53 (46.82)	33.99 (6.29)	4.61 (0.91)	34.45 (8.58)	7.49 (1.24)
11-OH-THC, μg/mL	6.05 (2.66)	17.18 (4.28)	8.55 (1.75)	1.03 (0.25)	3.59 (0.62)	1.87 (0.34)
CBD, ng/mL	1.53 (0.70)	5.07 (2.84)	1.20 (0.33)	3.35 (1.14)	123.83 (30.47)	30.68 (6.93)

THC, tetrahydrocannabinol; 11-OH-THC, 11-hydroxy-Δ9-THC; CBD, cannabidiol.

### Subjective drug effects

Self-reported intoxication significantly increased from pre- to post-cannabis use in both groups (*F*_1,51_ = 17.15, *p* < 0.001; Fig. 1a). There was also a significant time by concentrate interaction (*F*_1,102_ = 63.98, *p* < 0.001; Fig. 1a), with those in the THC-dominant concentrate group reporting a sharp increase in immediate intoxication levels (*t*_27_ = -13.90, *p* < 0.001) that significantly decreased over one hour (*t*_27_ = 4.45, *p* < 0.001). Ratings of intoxication in the CBD-dominant concentrate condition were significantly different between pre-use and immediate post-use (*t*_25_ = -8.92, *p* < 0.001), and also decreased over the subsequent hour (*t*_25_ = 3.21, *p* < 0.01). The ARCI marijuana effect measure revealed a significant effect of time (*F*_2,102_ = 11.46, *p* < 0.001), as well as a time by concentrate group interaction (*F*_2,102_ = 8.57, *p* < 0.001; Fig. 1b). Specifically, ARCI scores increased significantly from pre-use to immediate post-use for THC-dominant users (*t*_27_ = -7.08, *p* < 0.001) to a larger extent than for CBD-dominant users (*t*_25_ = -2.42, *p* = 0.02). A significant difference between groups was seen immediately post-use for overall drug liking, with a higher rating in the THC concentrate group (*F*_1,51_ = 6.28, *p* = 0.01; Fig. 1c).

In terms of affect measures, there was an overall increase in elation in both conditions immediately post-administration (*F*_2,102_ = 9.05, *p* < 0.001; Fig. 2a), although elation spiked immediately post-use in the THC condition (*t*_28_ = -3.51, *p* < 0.01) to a higher degree than in the CBD condition (*t*_25_ = -2.46, *p* = 0.02). Additionally, there was a significant decrease in tension over time after concentrate use (*F*_2,102_ = 8.83, *p* < 0.001; Fig. 2b). This effect was only seen in the CBD-dominant condition, where overall ratings of tension decreased immediately post-use (*t*_25_ = 2.77, *p* = 0.01), and even further after one-hour (*t*_25_ = 3.79, *p* < 0.01). A difference between groups was seen with anxiety, revealing a significant decrease in anxiety immediately post CBD-dominant concentrate use (*t*_25_ = 2.40, *p* = 0.02). Note that pre-use tension did not significantly differ between groups (*p* = 0.06). THC-dominant concentrate use took an hour to show significant effects, but ultimately had a more robust effect on lowering anxiety (*t*_26_ = 3.09, *p* < 0.01; Fig. 2c). However, the THC-dominant group showed significantly increased paranoia (*t*_27_ = -2.83, *p* < 0.01; Fig. 2d) immediately post-administration, while no effect was seen in the CBD-dominant condition.

## Discussion

This study explored the acute subjective effects of two cannabis concentrate products, one a THC-dominant concentrate, containing 84.99% total THC and < 1% CBD, and the other, a CBD-dominant concentrate, containing 78.8% total CBD and 4.5% total THC. Expected results were seen in terms of plasma cannabinoid levels in each condition, as well as higher intoxication and drug reward within the THC-dominant condition. Feelings of elation increased significantly in both conditions immediately post-use and declined over the subsequent hour, while tension and anxiety decreased immediately post-use in the CBD-dominant conditions. Anxiety-reduction was also demonstrated in the THC-dominant condition, but with a later onset 1-h after use. Finally, an immediate increase in paranoia was seen in the THC-dominant condition, which began to resolve within the following hour.

Cannabinoid blood levels at the experimental session indicate compliance with assigned treatment group. Specifically, high blood-CBD and low blood-THC immediately post-use in participants in the CBD-dominant group suggests that those individuals used the CBD product, while higher blood-THC and lower blood-CBD levels immediately post-use among those in the THC-dominant group suggest that they used the THC product. The results in the THC-dominant group in the present study are similar to findings from our prior study^18^, in which individuals using 70% or 90% THC concentrate products demonstrated somewhat higher THC blood levels immediately post-use (320 ng/mL) compared to immediate-post use plasma-THC in the THC-dominant concentrate group in the present analysis (238.58 ng/mL). However, given that different and more potent products were used in our prior study, some variation in resulting blood levels is expected. It is also notable that in both the current study and in our prior work, cannabinoid blood levels (THC and CBD) peak immediately after cannabis use, but drop considerably one-hour later^18,43^. This suggests that across different forms of inhaled cannabis products, regular users experience a significant peak in cannabis blood levels immediately post-use which resolves over the course of the next hour.

In line with the pattern of plasma cannabinoid results noted above, intoxication levels in the present study also increased immediately after concentrate use and decreased over the next hour. Intoxication displayed a significant increase in the CBD-dominant condition, likely due to the fact that there was THC present in the concentrate (although at a relatively low amount). Note that the presence of some THC is typical of CBD concentrate products on the market. Blood data in the CBD-dominant condition showed much lower levels of THC post-use, but the slight acute intoxication effect we observed is still reasonable to expect. Positive mood effects (elation) also significantly increased immediately post-use in both THC and CBD-dominant conditions and decreased significantly one hour later in both groups. Although still above baseline, these scores point to a relatively short-lived mood-enhancing effect of cannabis^44^.

Despite increased positive mood in both groups, the THC-dominant group demonstrated significantly more drug intoxication and rewarding effects (i.e. ARCI, drug liking) immediately post-use compared to the CBD-dominant group. This pattern may be explained by the amount of THC present in the CBD-dominant product being insufficient to produce the euphoric high that regular cannabis users may seek. This coincides with several studies that suggest dose-dependent effects of desirable, euphoric feelings induced by cannabis and THC products^11,12,45,46^. Of note, these mood enhancements can be associated with higher risks associated with acute and long-term use, such as impaired judgment and abuse liability.

A difference in groups was also observed in the measure of negative affective states. Specifically, we demonstrated a significant decrease in both anxiety and tension immediately post-use in the CBD-dominant concentrate group which, although qualitatively small, is consistent with the acute anxiolytic effects shown by a number of prior CBD studies^24,25,47–49^. This finding in conjunction with the existing literature supports the further exploration of hemp-based CBD (ideally taken in a lower-risk form, such as orally) as a potential alternative treatment for acute anxiety, although tightly-controlled human studies in this area are still warranted. In contrast, individuals in the THC-dominant concentrate group did not report significant changes in anxiety or tension immediately post-use, but a significant reduction in anxiety from baseline did emerge at one-hour post-use. This is notable as human studies generally show an anxiogenic acute effect of THC, especially in high concentrations^50^.

However, some subjective data do show a preference for THC or whole cannabis products in the context of self-medicating for affect issues, with few reports of undesirable effects^50,51^. Furthermore, anxiogenic effects induced by more typically seen in individuals who fall into the category of infrequent or non-users, rather than those who use cannabis or THC products on a more regular basis^25,50^. Importantly, anxiety and tension levels were low overall in the study and do not reflect clinical levels of negative affect. However, findings add to our understanding of the anxiolytic potential of cannabinoids and suggest that additional research is needed to explore the anxiolytic or anxiogenic effects at varying doses, using various THC:CBD ratios, and in among individuals with different types of cannabis use profiles (e.g., regular users, infrequent users, etc.). Future studies should also explore whether adding specific levels of CBD to THC-based concentrates results in a reduction of negative effects in users of such high potency products, as seen in prior studies using different products or methods of administration^28^.

The THC-dominant group reported significant increases in paranoia immediately post-administration, which resolved one hour later. Acute effects of paranoia following THC use are often noted in subjective outcomes within cannabis studies^11,13,52^. However, prior evidence regarding the duration of the effect is lacking. The current study suggests that while paranoia is an undesirable effect, negative effects of THC concentrates such as this are relatively short-lived. It is important to consider this as a dynamic process, and that the duration of positive, negative, and intoxication effects may tend to vary across individuals^11^.

Finally, in the 5 days prior to the van session, participants’ alcohol and cannabis use patterns were similar across groups and similar to their baseline patterns. These data support the notion that groups were similar on their cannabis use behavior and that participants continued their normal behavior leading up to the experimental appointments, which highlights the naturalistic element of the study design and may serve to bolster generalizability of these findings.

Limitations of the current study include the fact that, due to federal restrictions on legal-market cannabis research, the study design is observational and involves ad libitum administration of cannabis products rather than standardized or controlled cannabis doses. Future studies would also benefit from the use of precise matching of cannabinoid content and a placebo control cannabis product, however such products do not currently exist on the cannabis market. Although future studies would ideally test the acute effects of cannabis concentrates in controlled laboratory settings, tightly-controlled designs such as clinical trials are not currently permitted for these products due to federal restrictions on cannabis research. In addition, we did not collect data on drug expectancies regarding the effects of THC or CBD. Significant data suggests that drug expectancies can impact acute experiences following cannabis use^53,54^. Future studies would ideally include a measurement of drug expectancies prior to acute drug administration sessions. Finally, as the CBD-dominant concentrate did contain very low amounts of THC, the effects reported here (e.g. low levels of intoxication and other differences in subjective effects in the CBD-dominant group) may be influenced to some extent by THC present in the product. Despite these limitations, the study also has several notable strengths. In particular, the inclusion of legal-market concentrates, namely CBD-dominant concentrate products, is extremely novel, as these products have not been explored in any laboratory research to date. In addition, our focus on differential effects of THC and CBD may have implications regarding the use of CBD to mitigate harms associated with consumption of THC and/or in users of high THC products more generally. Furthermore, the naturalistic administration aspect of the study provides more generalizable insight into how both THC and CBD may impact individual users in the context of their typical use patterns.

Overall, these results support literature regarding the short-term effects of CBD in reducing anxiety and tension, particularly among users of THC-based cannabis concentrates. Additionally, this research supports existing literature regarding intoxicating and negative psychoactive effects of THC, such as acutely increased paranoia. Ultimately, those in the CBD-dominant condition saw a reduction in tension and anxiety while also experiencing a lower intoxication effect compared to the THC-dominant condition. This pattern may be interpreted as a harm reduction effect of a CBD-dominant cannabis, which was associated with some level of intoxication, but overall decreased negative affect and without the accompanying negative effects of higher levels of THC in our study of regular cannabis concentrate users. These results point to a further need to explore the harm-reducing potential of CBD, which could confer beneficial effects (e.g., on mood and drug reward) to individuals who use high amounts of THC, and perhaps especially for those who use highly potent THC concentrate products.
